# Retraction: Identification and Characterization of Cells with Cancer Stem Cell Properties in Human Primary Lung Cancer Cell Lines

**DOI:** 10.1371/journal.pone.0232726

**Published:** 2020-04-29

**Authors:** The PLOS ONE Editors

Following the publication of this article [1], concerns were raised regarding the flow cytometry data reported in Fig 3:

- There are similarities in dot plot patterns between the right panel of Fig 3A and the right panel of Fig 3C. Although the plots are not exact duplicates, the similarities observed are not expected from plots derived from distinct datasets.

The original data underlying Fig 3 are no longer available and thus it is not possible to resolve the concerns pertaining to the reliability of the integrity of the published figure.

The Commission on Research Integrity at Oslo University Hospital and Institute of Clinical Medicine, University of Oslo has completed an investigation and concluded that there was evidence of scientific misconduct.

In light of the outstanding concerns with panels in Fig 3 that question the integrity of these data and the outcome of an internal institutional investigation, the *PLOS ONE* Editors retract this article.

PW, QG, ZS, EM, LM, and GG did not agree with the retraction. SS, MW, NACW, and GK did not respond to the retraction decision.
